# Clinical and Surgical Approach to Oral Papilloma in the Soft Palate: A Case Report

**DOI:** 10.1155/crid/8847872

**Published:** 2026-03-04

**Authors:** María Victoria Espinoza-Salcedo, Edward Henry Miranda-Gutierrez, Jhair Alexander Leon-Rodriguez, Jorge Luis Huarcaya-López, Otto Jhonny Ajalcriña-Hernández, Alexander Roger Espinoza-Salcedo, Jhohan Ismael Leon-Rodriguez

**Affiliations:** ^1^ Postgraduate School, Antenor Orrego Private University, Trujillo, Peru; ^2^ Department of Stomatology, Trujillo Regional Teaching Hospital, Trujillo, Peru; ^3^ School of Stomatology, Antenor Orrego Private University, Trujillo, Peru; ^4^ School of Stomatology, Cesar Vallejo University, Piura, Peru; ^5^ Faculty of Health Sciences, Cesar Vallejo University, Trujillo, Peru

**Keywords:** biopsy, case report, palate, papilloma, soft

## Abstract

**Background:**

Oral papilloma is a benign lesion caused by the human papillomavirus (HPV), characterized by an exophytic, vegetative, verrucous, or papular growth in the oral mucosa. This type of lesion can be located in various areas of the oral cavity and represents one of the main clinical manifestations associated with HPV infections. Early diagnosis is crucial to rule out malignant conditions and ensure appropriate treatment.

**Case Presentation:**

A 59‐year‐old male patient attended the Odontoestomatology Department at the Regional Teaching Hospital of Trujillo due to an asymptomatic growth in the right soft palate with a 2‐year history. Clinical examination revealed an exophytic lesion approximately 5 mm in diameter, with normal coloration, rough texture, and a pedunculated base. An excisional biopsy of the lesion was performed, and histopathological analysis confirmed the presence of oral papilloma with epithelial hyperplasia and koilocytes, confirming the benign nature of the lesion.

**Conclusion:**

Surgical excision proved to be an effective and definitive treatment for oral squamous papilloma of the soft palate. Early recognition and appropriate management are crucial to differentiate benign papillomatous lesions from potentially malignant oral pathologies and to prevent unnecessary complications.

## 1. Introduction

Oral papillomas are hyperplastic, verrucous, and papillomatous lesions affecting the epithelial cells of the skin and mucosa, caused by the human papillomavirus (HPV) [1]. The term “papilloma” originates from the Latin *papilla* (pustule) and the Greek suffix *oma* (tumor). These lesions are part of a heterogeneous group of DNA viruses known as *Papillomaviridae* [2, 3]. HPV is responsible for various clinical conditions and is recognized as an independent risk factor for both benign and malignant tumors [4].

In recent years, an increased incidence of HPV‐positive oropharyngeal cancers has been reported [5]. Approximately 10% of men and 3.6% of women are affected by oral HPV infections, with prevalence increasing with age [6]. Globally, the prevalence ranges from 9% to 13%, affecting an estimated 630 million people. Developing countries report significantly higher rates compared to developed nations. Epidemiological studies conducted in the United States and Northern Europe have demonstrated a substantial rise in the incidence of HPV‐related oropharyngeal squamous cell carcinoma, accounting for more than 60% of the affected population [7].

Oral papilloma is one of the most common oral lesions and is primarily associated with HPV Subtypes 6 and 11, which are identified as etiological agents in approximately 50% of cases [8]. Infection begins when a viral particle penetrates a basal epithelial cell (keratinocyte) through microlesions or superficial abrasions in the mucosa. Once inside, the virus can remain latent or undergo active replication. In most cases (80%), these infections are transient and resolve spontaneously within 12–24 months, likely due to the host’s immune response. However, in some cases, they may progress to malignant lesions [9].

HPV can persist in a latent state for years without clinical or histological changes, or it may induce benign papillomatous lesions or warts. These lesions typically consist of hyperplastic tissue with thickened epithelial spinous layers and increased capillary proliferation [10]. Clinically, benign papillomas are characterized by painless papillary or verrucous lesions with a pedunculated or sessile base and coloration ranging from whitish to pink. In contrast, premalignant or malignant lesions, such as leukoplakia or squamous cell carcinoma, exhibit more aggressive features [11].

Histologically, oral papillomas display parakeratosis, severe epithelial acanthosis, elongation and widening of rete ridges, and exophytic papillary growths. Cellular alterations include perinuclear vacuoles, enlarged irregular hyperchromatic nuclei, and binucleation. These features are typical of koilocytes, regarded as the “hallmark” of HPV infection [12].

Peru, as a developing country, faces significant public health challenges, with HPV being a prevalent concern [13, 14]. Various studies highlight that a large portion of the population is affected by this infection, underscoring the importance of implementing effective strategies for its diagnosis and treatment, particularly in the context of developing countries [15–17].

This case is relevant because it documents the clinical and surgical management of an oral squamous papilloma located in the soft palate of an older adult patient treated in a public teaching hospital in northern Peru. This report adds to the existing literature by highlighting the importance of early recognition, accurate histopathological diagnosis, and complete surgical excision to prevent unnecessary concern and to rule out lesions with malignant potential.

## 2. Case Presentation

**Figure 1:**
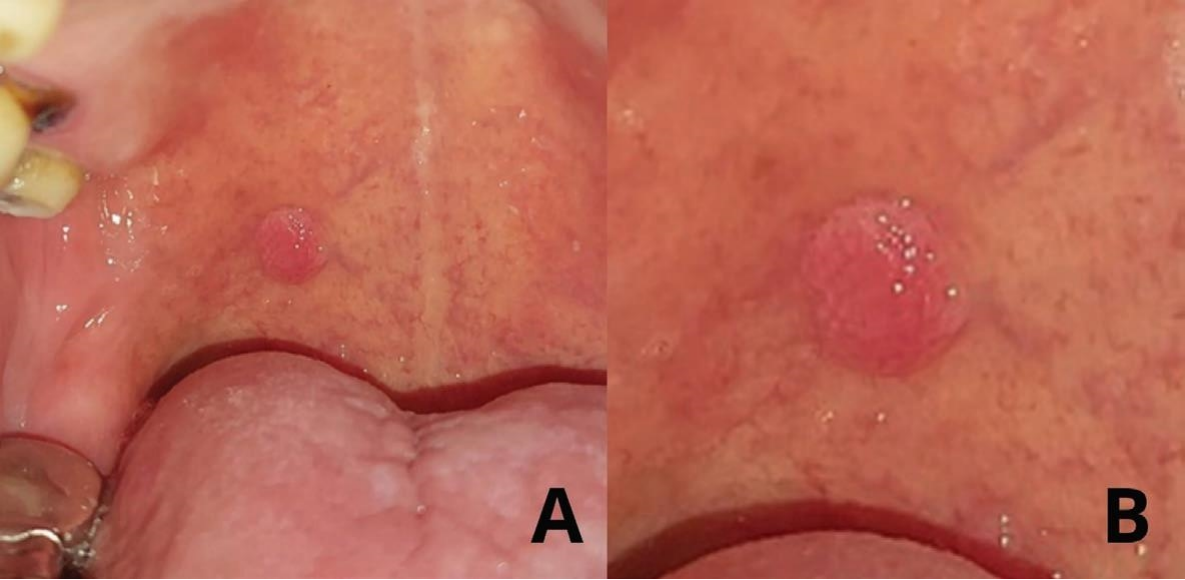
Clinical images of the lesion. (A) Clinical intraoral examination showing the lesion. (B) Enlarged view of the lesion on the soft palate.

A 59‐year‐old male patient presented to the Odontoestomatology Department at the Regional Teaching Hospital of Trujillo in northern Peru, with a localized growth on the right soft palate that had been evolving over 2 years. The lesion was asymptomatic but caused discomfort upon contact. Clinical intraoral examination revealed an exophytic lesion on the right soft palate, approximately 5 mm in diameter, with normal coloration, rough texture, and a pedunculated base (Figure 1a,b).

The patient reported no significant medical, family, or psychosocial history. He denied tobacco or alcohol use and had no known systemic conditions, immunosuppressive disorders, or previous oral lesions. No prior interventions or treatments had been performed for the current lesion during the 2‐year period in which it had been present. The sequence of clinical events during the patient’s care is summarized in Table 1.

**Table 1:** Timeline of clinical events during the patient’s care.

Time point	Clinical event
2 years before consultation	Patient noticed a small, asymptomatic growth on the right soft palate. No previous evaluation or treatment was sought during this period.
Day 0—clinical consultation	Intraoral examination revealed a 5‐mm exophytic, rough‐surfaced, pedunculated lesion on the right soft palate (Figure 1a,b). Differential diagnoses considered.
Day 0—biopsy and surgical procedure	Local anesthesia administered (2% lidocaine with epinephrine) (Figure 2a). Complete excisional biopsy performed with No. 15 blade, followed by simple suture closure (Figure 2b,c). Specimen fixed in 10% formalin for histopathologic study (Figure 2d,e).
Day 7—early postoperative follow‐up	Mild erythema consistent with normal healing; no pain, infection, or complications observed (Figure 3a,b). Sutures removed.
Within 2 weeks—histopathological report	Microscopy revealed epithelial acanthosis, epithelial hyperplasia, koilocytes, and mild dysplasia, confirming benign oral squamous papilloma (Figure 4a).
1‐month follow‐up	Complete mucosal healing with no recurrence, discomfort, or functional limitations. Patient clinically stable and asymptomatic (Figure 4b).
Current status	No recurrence reported; patient remains asymptomatic and lesion‐free.

### 2.1. Differential Diagnoses

The differential diagnosis included oral verruca vulgaris, condyloma, focal epithelial hyperplasia, and verrucous carcinoma due to the clinical similarities among these conditions. Diagnostic tools such as biopsy and molecular biology analysis are essential to accurately differentiate these lesions. Oral papillomas are most commonly found in areas such as the tongue, uvula, labial mucosa, frenulum, vermilion border, and soft palate, particularly when presenting as solitary lesions [4].

### 2.2. Diagnosis and Treatment

**Figure 2:**
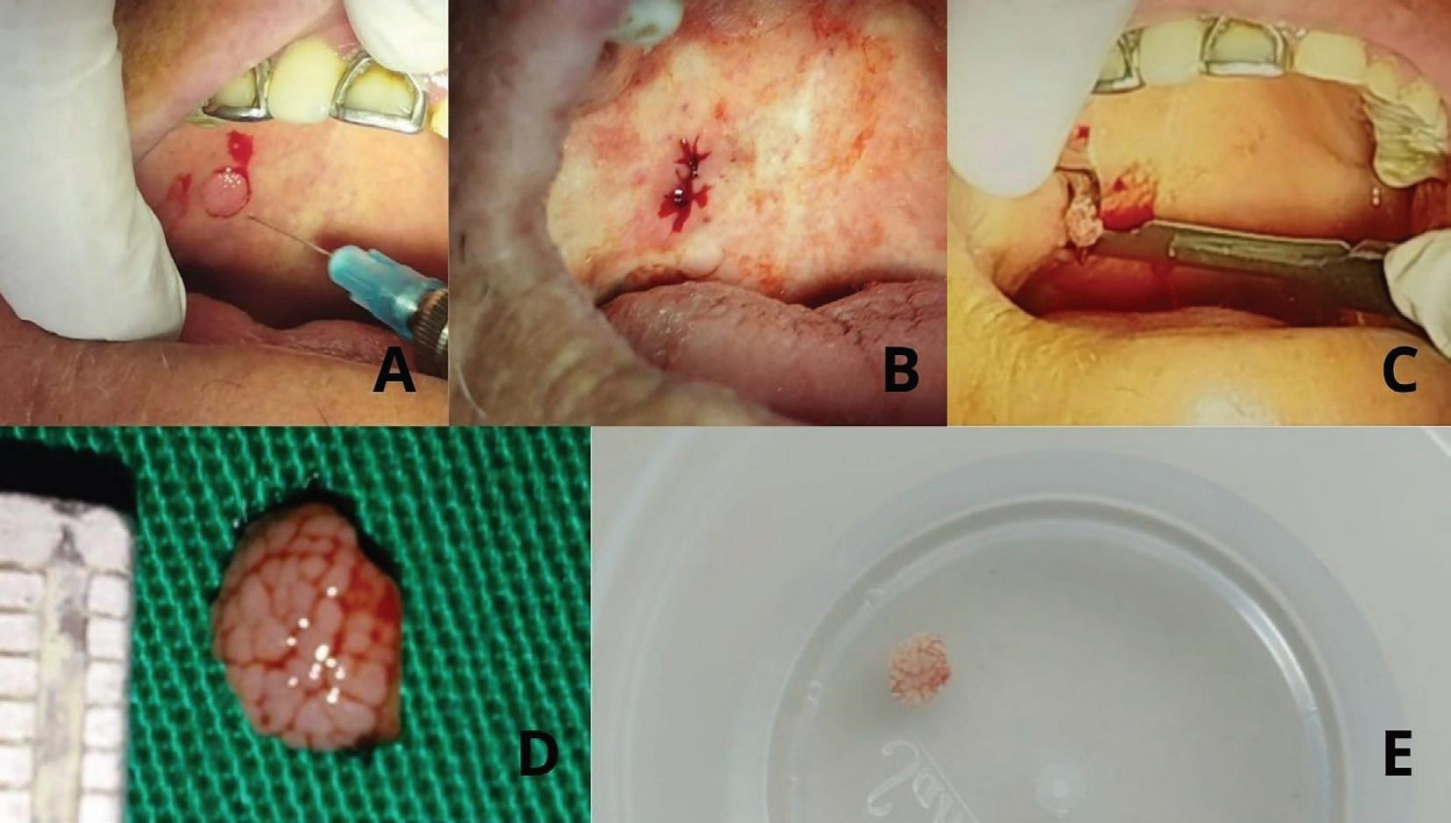
Step‐by‐step images of the biopsy procedure. (A) Anesthetic infiltration in the perilesional region. (B) Surgical excision procedure using a No. 15 scalpel blade. (C) Surgical closure with simple sutures. (D) Excisional biopsy process of the lesion. (E) Fixation of the surgical specimen in 10% formalin.

An excisional biopsy of the lesion was performed under local anesthesia using 2% lidocaine with epinephrine. A 30G, 21‐mm needle was used to infiltrate the anesthetic into the perilesional area (Figure 2a).

An excisional biopsy of the lesion was performed under local anesthesia using 2% lidocaine with 1:100,000 epinephrine (New Stetic S.A., glass cartridge). A 30G 21‐mm needle (Nipro Medical) was used to infiltrate the anesthetic into the perilesional area (Figure 2a).

The lesion was dissected using a No. 15 scalpel blade after securing it with a surgical forceps (Figure 2b). After achieving hemostasis, the tissue edges were approximated and closed with simple sutures (Figure 2c). The surgical specimen was fixed in 10% formalin and sent to the pathology laboratory for histopathological evaluation (Figure 2d,e).

**Figure 3:**
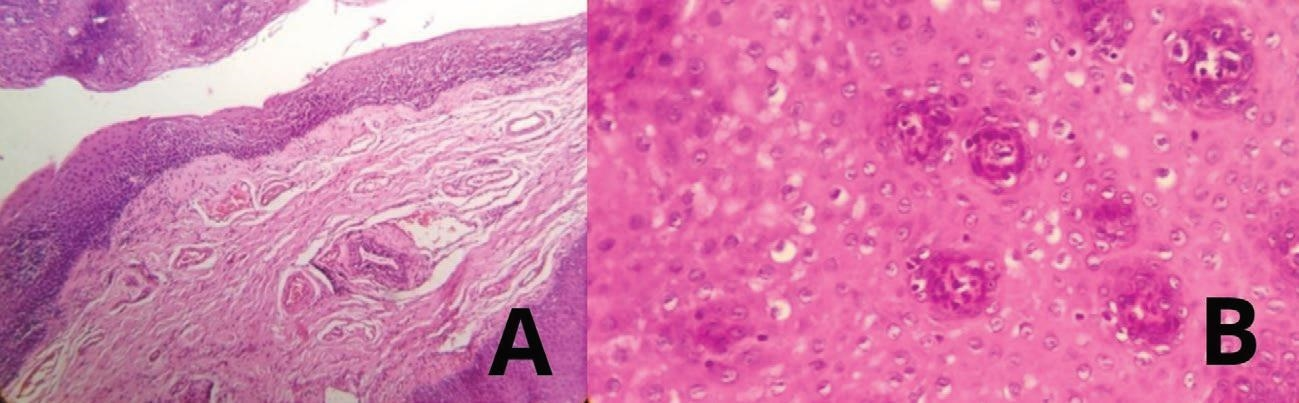
Histological images of the lesion. (A) Microphotograph (4×) showing epithelial acanthosis. (B) Microphotograph (40×) highlighting the presence of koilocytes.

Histopathological analysis, conducted in the Microbiology Area of the Regional Teaching Hospital of Trujillo, using an Olympus CX41RF microscope, revealed widening of epithelial projections due to acanthosis (Figure 3a), with the presence of multiple koilocytes and mild epithelial dysplasia. These findings confirmed the diagnosis of papilloma with epithelial hyperplasia and the benign nature of the lesion (Figure 3b).

No significant diagnostic challenges were encountered, although the clinical similarity to other papillomatous lesions required histopathological confirmation. The prognosis was excellent, with a very low risk of recurrence following complete surgical excision.

The treatment for lesions associated with HPV is primarily surgical, as they do not respond to topical or systemic pharmacological therapies. Surgical options include cold blade scalpel, quantum resonance scalpel, or laser excision, all of which allow for adequate specimen collection for histological examination.

**Figure 4:**
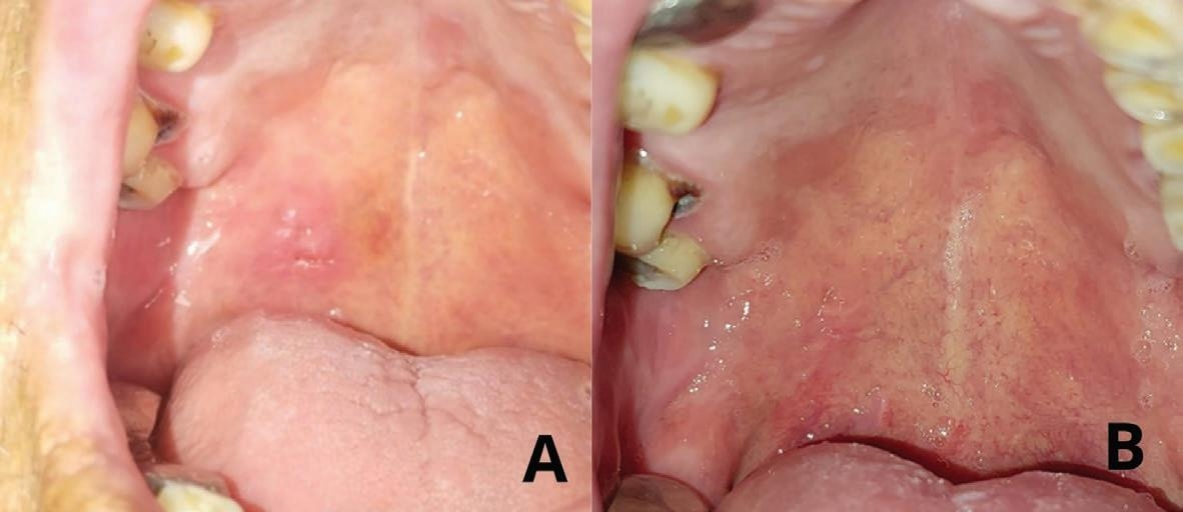
Postoperative clinical images. (A) Healing progression 7 days postoperatively. (B) Follow‐up at 1 month showing complete healing of the surgical site with no signs of recurrence or complications.

In this case, the treatment involved the surgical resection of the lesion. Local anesthesia was infiltrated into the affected area, the lesion was completely excised, and after ensuring hemostasis, the wound was closed with simple sutures. At 7 days postprocedure, a follow‐up examination was performed, revealing mild redness consistent with the normal healing process (Figure 4a).

At the one‐month follow‐up, the surgical site exhibited complete healing with no evidence of recurrence or complications. The patient reported no discomfort or functional impairment, and the clinical examination confirmed the absence of residual or new lesions, indicating a successful treatment outcome (Figure 4b). No adverse events or postoperative complications were observed.

The patient expressed satisfaction with the esthetic and functional outcome of the procedure. He reported relief after receiving a definitive diagnosis, appreciated the clarity of the treatment plan, and expressed confidence after observing the successful postoperative healing.

## 3. Discussion

Papilloma‐type lesions are more common in older adults, although they can occur in individuals of any age and gender. These lesions are clinically characterized by exophytic masses with a protruding appearance, pink coloration, and a surface resembling a cauliflower. In many cases, these lesions are asymptomatic and remain latent, showing no response to pharmacological treatments. For this reason, excisional biopsy is considered the standard treatment, as it allows both the removal of the lesion and the collection of a sample for histopathological evaluation [1, 2].

Orenuga et al. [18] reported a case of a recurrent squamous papilloma in a 5‐year‐old pediatric patient, located on the hard palate. During the intraoral examination, a proliferative, painless lesion with a cauliflower‐like appearance and a pedunculated base was observed. Although benign, these types of lesions can raise concern due to their clinical resemblance to conditions such as exophytic carcinoma or condyloma acuminatum. Histopathological analysis confirmed the presence of epithelial acanthosis and koilocytes, characteristic findings of a squamous papilloma associated with HPV. This case highlights the importance of excisional biopsy as the treatment of choice for these lesions.

Another case reported by Darwish [19] reported a case of a squamous papilloma located on the soft palate of a 36‐year‐old male patient with a history of chronic smoking. The lesion, described as white, warty, feather‐like, and firm in consistency, had been present since the patient was 10 years old but remained asymptomatic. Surgical excisional biopsy revealed epithelial hyperplasia with finger‐like projections, hyperkeratosis, and a fibrovascular connective tissue core, confirming the diagnosis of squamous papilloma. The findings highlight the importance of routine histopathological examination and follow‐up to detect potential recurrence or malignant transformation. This case reinforces the role of surgical excision as the primary treatment modality for squamous papillomas.

Jaju et al. [20] reported the case of a 25‐year‐old female with a 6‐month history of a slow‐growing, painless lesion on the hard palate. The lesion was exophytic, sessile, pink, with a pebbled surface, and soft in consistency. Verrucous growths were also observed on the patient’s left index finger and arms, suggesting possible autoinoculation. Submandibular lymph nodes were tender, and excisional biopsy confirmed the diagnosis of squamous papilloma, revealing stratified squamous epithelium with hyperkeratosis and koilocytes. The patient remained lesion‐free during a 1‐year follow‐up, demonstrating the efficacy of surgical excision as treatment.

Recent studies have emphasized that oral papillomas are often associated with the HPV, mainly Subtypes 6 and 11, which are responsible for up to 50% of cases. The infection typically occurs following minor abrasions in the mucosa, allowing the virus to enter basal keratinocytes. While most HPV infections are transient, remaining latent or self‐limiting, delayed diagnosis and management may increase the risk of progression to premalignant or malignant lesions [7, 8].

In general, the histological characteristics of oral papillomas include the presence of acanthosis, elongation of epithelial ridges, and koilocytes, considered the “hallmark” of HPV. These features align with findings reported in international literature, where clinical and histopathological findings have been critical in establishing the benign nature of these lesions. Differential diagnoses, such as condylomas, verrucae vulgaris, and verrucous carcinoma, should be considered due to their similar clinical characteristics [10].

Finally, surgical treatment is considered the gold standard for these lesions, with techniques ranging from conventional scalpel excision to laser devices. Studies have demonstrated that surgical excision not only allows for a definitive diagnosis but also ensures complete resolution with minimal risk of recurrence. [12] The integration of advanced diagnostic techniques, such as molecular biology, may further optimize the management of these lesions in the future.

Strengths of this case include the clear clinical presentation, the step‐by‐step documentation of the surgical procedure, and the confirmatory histopathological analysis, which allowed a precise diagnosis. However, this report has certain limitations, such as the absence of HPV genotyping, which would have provided additional etiological detail, and the lack of long‐term follow‐up beyond 1 month to fully assess the risk of recurrence. As a single case, the findings cannot be generalized, although they offer valuable clinical insight into the management of oral papillomas.

The key lesson from this case is that early recognition and complete surgical excision of small papillomatous lesions in the soft palate allow rapid resolution, prevent diagnostic uncertainty, and effectively rule out lesions with malignant potential. Careful clinical evaluation supported by histopathology remains essential for achieving optimal patient outcomes.

## 4. Conclusion

This case report highlights the essential role of dental surgeons in the diagnosis and successful management of oral papillomas, particularly when these lesions occur in anatomically complex regions such as the soft palate. The patient in this case achieved complete resolution of the lesion through surgical excision, demonstrating the effectiveness of this therapeutic approach. Early diagnosis is crucial to differentiate papillomas from other oral pathologies with malignant potential, thereby preventing complications and ensuring optimal patient outcomes.

Understanding the histopathological characteristics and clinical behavior of these lesions enables timely and precise management by dental surgeons. Through comprehensive care and multidisciplinary collaboration, therapeutic outcomes can be optimized, and the quality of life of patients with oral papillomas can be improved.

## Author Contributions

**María Victoria Espinoza-Salcedo:** conceptualization (supporting), methodology (lead), investigation (lead), and data curation (lead). **Edward Henry Miranda-Gutierrez:** methodology (supporting), writing—original draft (lead), visualization (lead), supervision (lead), project administration (lead), and funding acquisition (lead). **Jhair Alexander Leon-Rodriguez:** methodology (supporting), writing—review and editing (lead), and project administration (equal). **Jorge Luis Huarcaya-López:** investigation (supporting), writing—original draft (supporting), visualization (supporting), supervision (equal), and project administration (equal). **Otto Jhonny Ajalcriña-Hernández:** investigation (supporting), writing—original draft (supporting), visualization (supporting), supervision (equal), and project administration (equal). **Alexander Roger Espinoza-Salcedo:** conceptualization (supporting), investigation (supporting), writing—original draft (supporting), and visualization (supporting). **Jhohan Ismael Leon-Rodriguez:** methodology (supporting), writing—review and editing (equal), and project administration (equal).

## Funding

This research did not receive any specific grant from funding agencies in the public, commercial, or not‐for‐profit sectors.

## Disclosure

It was conducted as part of the authors’ academic responsibilities at the Department of Stomatology, Trujillo Regional Teaching Hospital, Trujillo, Peru.

## Ethics Statement

Written informed consent was obtained from the patient for publication of all clinical information and images. This case was conducted in accordance with the ethical principles of the Declaration of Helsinki.

## Conflicts of Interest

The authors declare no conflicts of interest.

## Data Availability

The data that support the findings of this study are available on request from the corresponding author. The data are not publicly available due to privacy or ethical restrictions.
